# Conditional knockout of REST/NRSF in excitatory neurons reduces seizure susceptibility to chemical kindling

**DOI:** 10.3389/fncel.2023.1267609

**Published:** 2023-11-16

**Authors:** Giulia Natali, Caterina Michetti, Alicja Krawczun-Rygmaczewska, Thomas Floss, Fabrizia Cesca, Fabio Benfenati

**Affiliations:** ^1^Center for Synaptic Neuroscience and Technology, Istituto Italiano di Tecnologia, Genova, Italy; ^2^Department of Experimental Medicine, University of Genova, Genova, Italy; ^3^Department of Life Sciences, University of Trieste, Trieste, Italy; ^4^Helmholtz Zentrum München, Deutsches Forschungszentrum für Gesundheit und Umwelt, Neuherberg, Germany; ^5^IRCCS Ospedale Policlinico San Martino, Genova, Italy

**Keywords:** RE-1 silencing transcription factor, neuron-restrictive silencer factor, cell-specific knockout, epilepsy, pentylenetetrazol

## Abstract

The repressor element-1 silencing transcription factor/neuron-restrictive silencer factor (REST/NRSF) is an epigenetic master regulator that plays a crucial role during nervous system development and maturation. REST function was originally described during development, where it determines neuronal phenotype. However, recent studies showed that REST participates in several processes in the adult brain, including neuronal plasticity and epileptogenesis. In this regard, the relationships between REST and epilepsy are still controversial and need further investigation. As forebrain excitatory neurons are the common final pathway of seizure susceptibility, we investigated the role of REST in epilepsy by inducing REST conditional knockout (REST-cKO) specifically in excitatory neurons of the hippocampus. To target the excitatory neuronal population, we cloned the calcium/calmodulin-dependent protein kinase IIα minimal promoter upstream of Cre recombinase. After assessing the specificity of the promoter's expression, the transgenes were packaged in an engineered adeno-associated virus able to cross the blood–brain and blood–cerebrospinal fluid barriers and delivered in the lateral ventricles of 2-month-old REST^flox/flox^ mice to characterize, after 1 month, the cognitive phenotype and the seizure propensity. We show that REST-cKO mice display lower levels of anxiety in the light–dark test with respect to control mice but have unaltered motor, social, and cognitive profiles. The evaluation of the susceptibility to epileptic seizures showed that REST-cKO mice are more resistant to pentylenetetrazole-induced kindling but not to seizures induced by a single administration of the convulsant and show higher survival rates. Overall, these data suggest that the absence of REST in forebrain excitatory neurons decreases seizure susceptibility, pointing to a pro-epileptogenic role of the transcriptional repressor under conditions of pathological excitation/inhibition imbalance.

## 1 Introduction

The repressor element-1 (RE1) silencing transcription factor, also known as the neuron-restrictive silencer factor (REST/NRSF, henceforth referred to as REST), is an epigenetic master regulator that acts mainly by repressing transcription of a large cluster of target genes most of which are neuron-specific. Upon binding to a 21-base pair sequence called repressor element 1 (RE1), REST recruits and coordinates the action of several chromatin-packaging enzymes (Ooi and Wood, 2007). RE1 sequences reside in numerous neuronal genes encoding ion channels and neurotransmitter receptors and synthesizing enzymes and proteins involved in axonal guidance, synaptic vesicle trafficking, and fusion (Bruce et al., 2004). Therefore, the best-described role assigned to REST is to master the acquisition of the neuronal phenotype during development. Indeed, during neurogenesis, fluctuations in the levels of REST and its complex shape neuronal identity. REST levels are the highest in the nuclei of pluripotent stem cells. During the transition to neural progenitors, the REST mRNA level is kept constant although the protein level is dramatically decreased because of post-translational degradation. During the transition to mature neurons, REST detaches from the chromatin of neuronal genes and is concurrently transcriptionally repressed (Ballas et al., 2005). REST is also critical for the fine-tuning of neuronal network wiring and plasticity during development and adulthood. For example, it contributes to the chloride switch by modulating the transcription of potassium chloride transporter 2 (KCC2), fundamental for the maturation of GABAergic inhibitory transmission (Ganguly et al., 2001). Moreover, REST also regulates the N-methyl-D-aspartate receptor (NMDAR) subunit switch during post-natal development (Rodenas-Ruano et al., 2012).

At the circuit level, REST mediates homeostatic protection from network hyperexcitability by scaling down intrinsic excitability (Pozzi et al., 2013) and excitatory synaptic transmission (Pecoraro-Bisogni et al., 2018). In addition, REST scales up inhibitory synapses onto excitatory neurons, without affecting those on inhibitory neurons (Prestigio et al., 2021). The latter effect also involves positive transcriptional effects of REST on specific genes. A similar effect was recently shown in astrocytes, in which REST stimulates the transcription of the glutamate transporter GLT1, which in turn favors the membrane exposure of the potassium channel Kir4.1, boosting glutamate uptake and potassium buffering at the synapse and counteracting hyperexcitability induced by high-frequency synaptic and neuronal activity (Pajarillo et al., 2021; Centonze et al., 2023).

Due to its homeostatic transcriptional effects on neural genes controlling excitability and synaptic transmission, REST has been implicated in the pathogenesis of epilepsy, although conflicting results have been reported. In epilepsy, we not only observed morphological and functional alterations but also changes in the genetic pattern expressed by cells. In recent years, genome-wide analysis has found abnormal expression of many candidate genes for epileptogenesis implicated in inflammation, stress, synaptic transmission and plasticity, signal transduction, ion transport, and cell metabolism that can be interpreted as the result of a complex transcriptional dysregulation of the above-mentioned genes (Kobow and Blümcke, 2011). The first evidence that seizures can induce epigenetic changes in the brain comes from studies demonstrating the overexpression of REST after induction of epilepsy (Palm et al., 1998; Spencer et al., 2006). Whether REST dysregulation is the cause of hyperexcitability or represents an endogenous homeostatic response attempting to counteract hyperexcitability is still a debated issue.

Since the constitutive knockout of REST is embryonic lethal (Nechiporuk et al., 2016), the role of REST in epilepsy has been studied through conditional knockout (cKO) strategies. A cKO mouse model was initially generated to specifically delete REST in excitatory forebrain neurons and the impact of this deletion was studied in a kindling model of epileptogenesis. cKO mice had higher susceptibility to kindling, suggesting a protective role of REST against epilepsy (Hu et al., 2011). On the opposite, a reduced number of KA-induced seizures was observed when REST activity was reversibly inhibited by the hippocampal expression of a synthetic probe competing with the binding of the cofactor mSin3 to REST (Carminati et al., 2020). Similar results were found by McClelland et al. (2011), who reported that during kainic acid (KA)-induced status epilepticus, REST downregulates the expression of the hyperpolarization-activated cyclic nucleotide-gated ion channel (HCN1), making mice more vulnerable to seizures and thus contributing to the progression of epilepsy. Similarly, in a pentylenetetrazol (PTZ)-epilepsy model, mice bearing cKO of REST in all neurons displayed higher resistance to convulsions, higher PTZ lethal dose, increased survival, and alleviated epileptiform convulsions compared to control animals (Liu et al., 2012).

The reasons for these apparent discrepancies can be the different seizure models, the distinct neuronal populations targeted for the REST-cKO, the temporal window of REST downregulation, and the fact that REST might have different functions in the epileptogenesis phase and in the overt disease. Moreover, all previous studies used a conditional REST-KO model in which residual REST transcription is present. This issue was overcome by the generation of a newer REST cKO mouse in which no expression of REST and its truncated variants occurs (Nechiporuk et al., 2016). In addition, the subset of genes that are repressed by REST may also differ between physiological and pathological conditions. Indeed, it was found that evoked seizure activity greatly increased REST expression in the hippocampus, but only a very small percentage of REST target genes were repressed (McClelland et al., 2014), suggesting that REST dysregulation occurring under epileptogenic conditions may favor, rather than oppose, the development of epilepsy.

In this study, we investigated the role of REST in seizure propensity by using the floxed REST^*GTi*^ mouse line (Nechiporuk et al., 2016) in which REST was specifically deleted in adult forebrain excitatory neurons of adult mice. To this aim, we cloned the CaMKIIα minimal promoter upstream of the Cre recombinase in adeno-associated viral (AAV) vectors bearing an engineered viral capsid (PHP.eB) that provides improved transduction efficiency and ability to cross biological barriers. After confirming the specificity and penetrance of the engineered constructs, we investigated the behavioral phenotype and the seizure propensity of adult mice using both acute and chemical kindling paradigms. We found that REST-cKO mice are more resistant to PTZ-induced kindling showing higher survival rates from tonic-clonic seizures, suggesting that REST dysregulation triggered by hyperexcitability has a pro-epileptogenic role in forebrain excitatory neurons.

## 2 Materials and methods

### 2.1 Experimental animals

Wild-type C57BL/6J male mice were obtained from Charles River (Calco, Italy). Heterozygous GTinvREST mice (REST^GTi^ mice; Nechiporuk et al., 2016) were kindly provided by Gail Mandel (Portland, OR) and the German Gene Trap Consortium (GGTC-Partners). Animals were maintained on a C57BL/6J background and kept in homozygosity. Two females were housed with one male in standard Plexiglas cages (33 × 13 cm) under conditions of environmental enrichment. After 2 days of mating, male mice were withdrawn, and dams were housed individually in plexiglass cages and checked daily. Mice were weaned into cages of same-sex pairs maintained on a 12:12 h light/dark cycle at constant temperature (22 ± 1°C) and humidity (60 ± 10%), with a water and pellet diet (Mucedola, Settimo Milanese, Italy) *ad libitum*. All efforts were made to minimize suffering and reduce the number of animals used. All the experiments were carried out in accordance with the guidelines established by the European Community Council (Directive 2014/63/EU of 15 May 2014) and were approved by the Italian Ministry of Health (Authorization #558/2016-PR and #427/2021-PR). Only male mice were included in this study to avoid hormonal fluctuations in seizure susceptibility associated with the estrous cycle of female animals.

### 2.2 Plasmid cloning

The CaMKII minimal promoter was amplified by PCR from pLenti-CaMKII-hChR2-mCherry-WPRE using CaMKII-specific primers. The isolated promoter was digested with SpeI and NotI enzymes (Thermo Fisher Scientific, Waltham, MA) and cloned in pAAV-CAG-NLSx2-GFP (Chan et al., 2017), previously digested with the same enzymes to remove the CAG promoter.

To have a suitable control for the REST-cKO mice, we used a Cre deletion mutant (ΔCre) that is catalytically inactive (deletion at the level of the catalytic domain, residues 134-343). ΔCre and Cre sequences were amplified by PCR using specific primers from pLenti-PGK-ΔCre-GFP and pLenti-PGK-Cre-GFP (Kaeser et al., 2011), respectively. The isolated ΔCre sequence was digested with NotI and BamHI enzymes (Thermo Fisher Scientific, Waltham, MA) and cloned in the intermediate plasmid carrying the CaMKII promoter. Isolated Cre sequence was digested with NotI and PacI enzymes (Thermo Fisher Scientific, Waltham, MA) and cloned in the intermediate plasmid carrying the CaMKII promoter, and the additional PacI enzymes added cloning overlapping oligos. For all the cloning steps, plasmids were transformed into Stbl3 cells, and positive colonies were identified by DNA sequencing. The primers used were the following: CaMKII-FW: 5′-atccactagtacttgtggactaagtttgttcg-3′, CaMKII-RV: 5′-atccgcggccgcgctgcccccagaactagg-3′, ΔCre and Cre-FW: 5′-atccgcggccgccaccatggtgaagcgacc-3′, ΔCre-RV: 5′-atccggatccctacttacggattcgccgc-3′, Cre-RV: 5′-atccttaattaactaatcgccatcttccagcag-3′, Oligo containing Pac1-FW: 5′-gatccatatgttaattaaggcgcgcccaattg-3′, and Oligo containing Pac1-RV: 5′-gatccaattgggcgcgccttaattaacatatg-3′.

### 2.3 AAV production

Before starting virus production, we checked for the integrity of the ITRs by digesting the recombinant plasmids with the SmaI enzyme (Promega, Madison, Wisconsin), which has its restriction sites inside the AAV2 packaging signals (ITRs) sequences. AAV1/2 expressing pAAV-CaMKII-NLSx2-GFP were generated as previously described (McClure et al., 2011). Briefly, human embryonic kidney (HEK) 293T cells were co-transfected with the AAV plasmid containing the recombinant expression cassette flanked by ITRs, together with the plasmids pRV1, pH21, and pHelper using the Ca^2+^ phosphate method. After 48 h of transfection, cells were harvested and lysed. Viruses were purified over heparin columns (GE HealthCare Life Science, Milano, Italy), titrated at concentrations ranging from 1 x 10^11^ to 1 x 10^12^ vector genomes (vg)/ml, and used at a multiplicity of infection of 10,000. The efficiency of infection, estimated by counting neurons expressing the reporter GFP with respect to the total number of cells stained with DAPI, ranged between 70 and 90%. pAAV-CaMKII-NLS-GFP-ΔCre and pAAV-CaMKII-NLS-GFP-Cre were packaged into PHP.eB by Vector Biolabs (Malvern, PA).

### 2.4 Stereotaxic injections

Mice were anesthetized by the inhalation of isoflurane (0.8–1.5 L/min, 1–3% concentrated) and placed in the stereotaxic frame. We measured the x and y coordinates of bregma and marked the injection site (cortex: AP−1.46 mm, L +3 mm, DV +1.5 mm; lateral ventricles: AP−0.5 mm, L +/-1.5 mm, DV−1.5 mm). Then, 10^9^ vg were delivered at a controlled speed (cortex: 1 at 0.1 μl/min; lateral ventricles: 1-3 μl/ventricle at 0.1-0.0.3 μl/min) with a Hamilton syringe (Hamilton, Reno, NV) and the glass capillary was held in position for additional 5 min to avoid virus backflow. At the end of the procedure, we sutured the surgical wound and administered analgesics (ketoprofen 1–0.5 mg/kg, dexamethasone 5 mg/kg) through intraperitoneal injection.

### 2.5 Immunofluorescence

Mice that were previously injected with either AAV1/2 CaMKII-NLSx2-GFP or PHP.eB CaMKII-NLS-GFP-ΔCre were deeply anesthetized with an intraperitoneal injection of a mixture of 100 mg/kg ketamine and 10 mg/kg xylazine and transcardially perfused with ice-cold phosphate-buffered saline (PBS), followed by 4% paraformaldehyde in PBS. After perfusion, brains were collected and post-fixed in the same fixative solution overnight at 4°C. After several washes in PBS, brains were cryoprotected by immersion in 30% sucrose solutions and cut into 30 μm sections with a Vibratome (Leica, Wetzlar, Germany). Sections were stored at−20°C in a solution containing 30% ethylene glycol and 20% glycerol in 0.1 M phosphate buffer. Sections were washed in PBS and processed for free-floating immunofluorescence. After a blocking step with a solution containing 0.1% Triton X-100 and 10% normal goat serum (NGS), sections were incubated overnight at 4°C with a combination of the following primary antibodies: guinea pig anti-green fluorescent protein (GFP; 1:500, Synaptic Systems), rabbit anti-green fluorescent protein (GFP; 1:500, Synaptic Systems), mouse anti-CaMKII (1:100, Cell Signaling, Malvern, PA), mouse anti-glutamate decarboxylase 67 (GAD67; 1:500, Merck), guinea pig anti-glial fibrillary acidic protein (GFAP; 1:500, Synaptic Systems), guinea pig anti-ionized calcium-binding adapter molecule 1 (Iba1; 1:500, Synaptic Systems), and mouse anti-neuronal nuclear antigen (NeuN; 1:500, Merck, Milano, Italy). Sections were then washed in PBS (3 x 10 min) and incubated for 2 h at room temperature with anti-guinea pig Alexa Fluor 488 and 647, anti-rabbit Alexa Fluor 488, and anti-mouse Alexa Fluor 568. Sections were washed in PBS (3 x 10 min), mounted on glass slides, and observed with a Leica SP8 laser-scanning confocal microscope (Leica, Wetzlar, Germany).

### 2.6 Analysis of transduction specificity and virus spread

We imaged brain slices with a 63X oil immersion objective. For each slice, we acquired serial optical sections, at the optimized z-step size, spanning over the total slice depth. GFP positive (GFP+) and cell-type specific positive cells were manually counted with the ImageJ software. Two parameters were then evaluated: the specificity of minimal promoter expression, expressed as the percentage of cell-type specific-GFP+ cells on the overall GFP+ cells, and the penetrance, expressed as the percentage of cell-type specific-GFP+ cells on the overall selected population. To determine the virus spread along the brain rostro-caudal axis, we imaged brain slices with a 20X objective. We acquired optical sections at three distances from bregma, at the optimized z-step size, spanning over the total slice depth. Single acquisitions were merged to produce a large image representing the entire brain slice. We further characterized the virus spread in the main transduced brain areas (hippocampus, primary motor cortex, and primary somatosensory cortex). Images were acquired every 100 μm with a 20X objective and automatically analyzed with a MATLAB script to quantify the ratio between GFP+ cell volume over the NeuN+.

### 2.7 RT-qPCR assessment of REST cKO

To verify the efficiency of our cKO system, we quantified the mRNA of REST coding exon 2-3 of mice injected with either PHP.eB CaMKII-NLS-GFP-ΔCre or PHP.eB CaMKII-NLS-GFP-Cre in both lateral ventricles. RNA was isolated from the mouse hippocampi with the phenol/chloroform extraction technique. The complementary cDNAs were synthesized by reverse transcription of 1 mg of RNA using the SuperScript IV Reverse Transcriptase (Thermo Fisher Scientific) with an oligo-dT primer according to the manufacturer's instructions. cDNAs were then used as templates for RT-qPCR using a C1000 Touch^TM^ Thermal Cycler (BioRad) on a CFX96^TM^ Real-Time System following the manufacturer's instructions. The expression of REST mRNA was quantified using the DDCT method, and data were normalized on the efficiency of transduction using the GFP mRNA levels. The primers used were the following: REST exon 2-3-FW: 5′-agaacgcccgtataaatg-3′, REST exon 2-3-RV: 5′-atggcttctcacctgaatg-3′, GFP-FW: 5′-agtccgccctgagcaaaga-3′, and GFP-RV: 5′-tccagcaggaccatgtgatc-3′.

### 2.8 Behavioral analysis

In the study, 3-month-old male mice that were previously injected with either PHP.eB CaMKII-NLS-GFP-ΔCre or PHP.eB CaMKII-NLS-GFP-Cre in both lateral ventricles were subjected to the following behavioral tests after 4 weeks from the stereotaxic procedure.

#### 2.8.1 Open field

General exploratory locomotion was tested in an open-field arena. Mice were placed at the center of the arena and let move freely for 30 min. The sessions were videotaped and traveled distance and mean speed were quantified.

#### 2.8.2 Light–dark box

A box partitioned into a dark and a light compartment was used for this test. At the beginning of each trial, mice were placed in the light compartment, facing away from the partition, and let freely move for 5 min. Time spent in each compartment in the first 5 min of the test and the latency to enter the dark compartment were quantified.

#### 2.8.3 Male–female social interaction

An unfamiliar estrus C57BL/6J female was inserted in the male cage to permit 3 min of free interaction. The vaginal estrous condition of each stimulus female was assessed as in Rugh (1991), and only females in estrus were selected as stimuli. All male subjects were sexually naïve at the time of testing. The sessions were videotaped and social sniffing (sum of nose-to-nose, nose-to-anogenital, and nose-to-other body regions sniffing) was scored.

#### 2.8.4 Three chambers

A box partitioned into three compartments, divided by retractable doorways, was used, and the test was conducted as in Yang et al. (2011). The test began with a habituation phase where mice were placed in the central compartment, with the doorways closed, for 10 min. Then, doorways were opened, and mice were let freely explore the three compartments for an additional 10 min. After the habituation phase, mice were briefly restricted to the central compartment while two empty cups were placed in each of the side chambers. An unfamiliar C57BL6/J mouse, previously habituated to the enclosure, was placed under one of the two empty cups. The side containing the empty cup and the mouse was alternated between the left and right chambers across tested mice. When both stimuli were set, the doorways were opened, and mice were allowed to explore the side compartments for 10 min (sociability phase). In the last phase of the test, mice were briefly restricted to the central compartment and a novel C57BL6/J mouse was placed under the empty cup. Doorways were opened, and mice were allowed to explore the side compartments for 10 min (novelty phase). Sociability and social novelty preferences were analyzed.

#### 2.8.5 Contextual fear conditioning

The test was performed in a box equipped with a grid calibrated to deliver foot shocks (Med Associated Inc.). The test consisted of three phases. In the conditioning phase (10 min), mice were placed in the box, and, after 2 min, 3 footshocks (2 s, 0.5 mA) were delivered with intertrial intervals (ITI) of 2 min. Contextual conditioning was tested 24 h later in the same chamber but without foot shocks to evaluate fear recall. Mice were placed in the chamber for 2 min, and freezing time was quantified. At the end of the session, animals were put back in the home cage for 1 h and then subjected to a novel context. In the novel context phase, the chamber environment was modified. Mice were placed in the altered chamber for 2 min, and freezing time was quantified.

### 2.9 Seizure induction

Mice that were previously injected in both lateral ventricles with either PHP.eB CaMKII-NLS-GFP-ΔCre or PHP.eB CaMKII-NLS-GFP-Cre were subjected to acute seizure and chemical kindling induction protocols.

#### 2.9.1 Acute generalized seizure

Pentylenetetrazol (PTZ, Merk) was dissolved in saline and administered i.p. at a dose of 65 mg/kg. Immediately after the PTZ injection, mice were single-housed and videotaped for 30 min or until death occurred. The following parameters were evaluated: latency and occurrence of the first myoclonic twitch (sudden, brief, and involuntary twitch of a single muscle or a group of muscles), latency and occurrence of death, and seizure duration. The average seizure score was assigned as follows: 0, normal behavior; 1, immobility; 2, twitch; 3, tail held up stiffly (Straub's tail); 4, mild seizure, the animal falls on its side; 5, severe seizure, the animal rushes and jumps; and 6, death (Shimada and Yamagata, 2018).

#### 2.9.2 Kindling

To select the proper PTZ dose to be administered for the kindling protocol, we tested animals with the following doses: 45, 50, 55, and 60 mg/kg. In the kindling protocol, single-caged 3-month-old mice that were previously injected with either PHP.eB CaMKII-NLS-GFP-ΔCre or PHP.eB CaMKII-NLS-GFP-Cre were intraperitoneally injected with PTZ 50 mg/kg on alternate days for 2 weeks. After each injection, mice were videotaped for 15 min or until death occurred. To evaluate the progression of the seizure phenotype during kindling, we evaluated the following parameters: the average score of each experimental group through the injection days, the occurrence of every single score among each group, the occurrence of seizure and death events, the probability of survival, and the average number of seizures experienced by each animal during the protocol.

### 2.10 Statistics

Data are shown as means ± standard deviation (SD) of the mean. The normal distribution of experimental data was assessed using the Shapiro–Wilk normality test. To compare two sample groups, either Student's *t*-test (normal distribution) or the Mann–Whitney *U*-test (non-normal distribution) was used. To compare more than two normally distributed experimental groups, one- or two-way ANOVA followed by Tukey's multiple comparison test were used. Contingency analysis was performed by using Fisher's exact test. The comparison of survival curves was performed with the Mantel-Cox test. A *p*-value of < 0.05 was considered statistically significant. All statistical data analysis and data representation were performed using GraphPad Prism 8 software (GraphPad Software Inc., San Diego, CA, USA), while data organization was performed with Microsoft Excel (Microsoft, Redmond, Washington, USA).

## 3 Results

### 3.1 Building and characterization of rAAV probes for *in vivo* targeting of excitatory neurons

**Figure 1:**
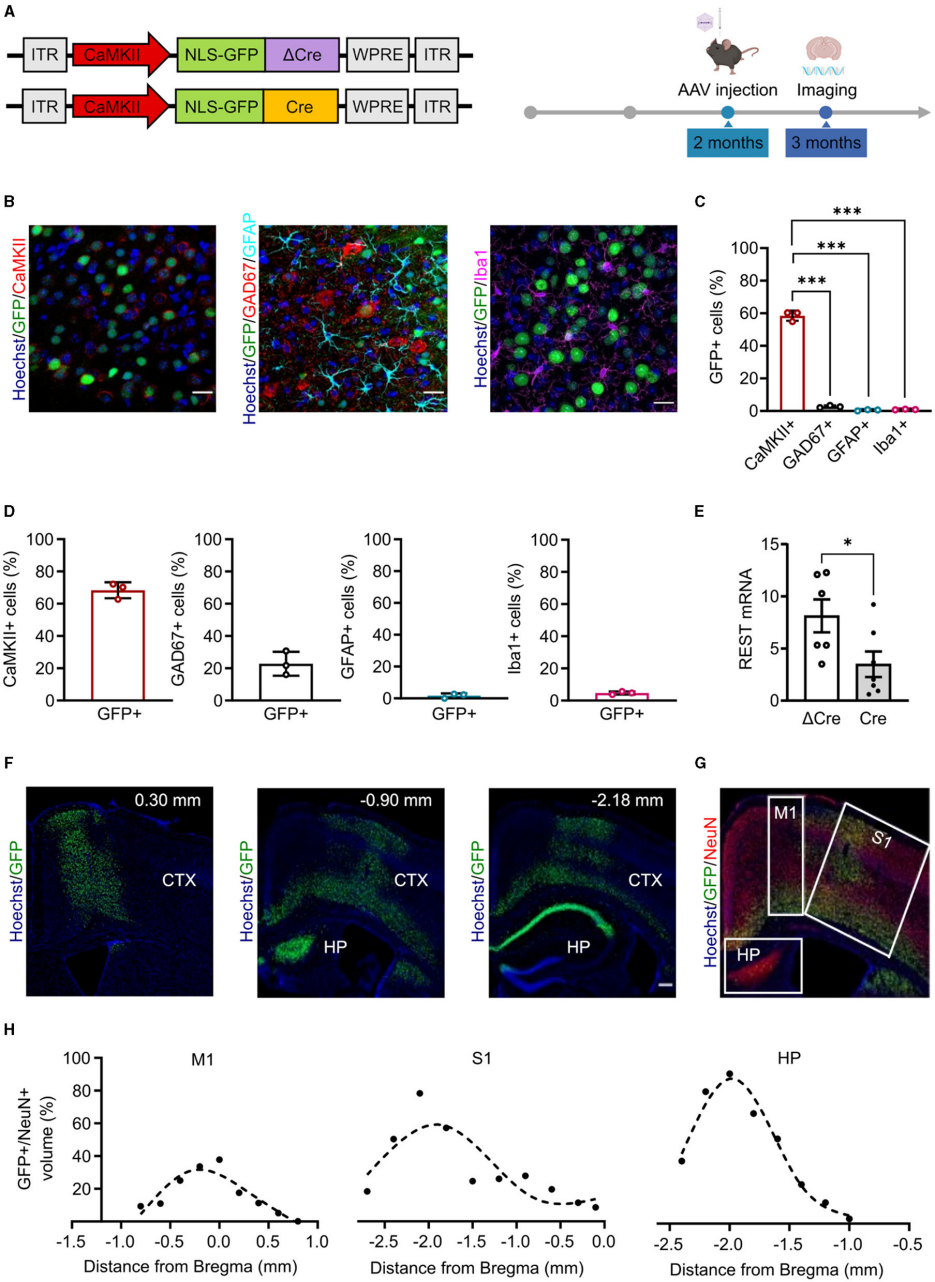
Minimal CaMKII promoter specifically targets forebrain excitatory neurons in the cortex and hippocampus. **(A)** *Left:* Scheme of the AAV constructs used in the study. ITR, inverted terminal repeats; CaMKII, calcium/calmodulin-dependent protein kinase II; NLS-GFP, nucleus-localized green fluorescent protein; ΔCre, inactive Cre recombinase; Cre, active Cre recombinase; WPRE, woodchuck hepatitis virus post-transcriptional regulatory element. *Right*: Timeline of the AAV injection and imaging experiments. **(B)** Representative images of brain cortical slices from mice injected in the cortex with AAV1/2 CaMKII-NLSx2-GFP. The nuclear stain with Hoechst (blue) and the GFP immunoreactivity (green) are associated with staining for CaMKII (red; *left*), GAD67 (red) and GFAP (cyan) (*middle*), and Iba1 (magenta; *right*). Scale bars, 20 μm. **(C)** Promoter expression specificity calculated as the ratio of the number of CaMKII+/GFP+ cells (red), GAD67+/GFP+ cells (black), GFAP+/GFP+ cells (cyan), and Iba1+/GFP+ cells (magenta) over the total number of GFP+ cells. Data are expressed as means ± SD. ***p < 0.001, one-way ANOVA/Tukey's tests. **(D)** Promoter penetrance is expressed as the ratio of the number of GFP+ cells over the total CaMKII+ (red histogram), GAD67+ (black histogram), GFAP+ (cyan histogram), and Iba1+ (magenta histogram) cells. Data are expressed as means ± SD (*n* = 3 mice, 5 slices per mouse). **(E)** RT-PCR quantification of REST mRNA levels in the hippocampus of ΔCre and Cre mice. The REST mRNA levels were normalized on the GFP expression. Data are expressed as means ± SD. *p < 0.05, Student's *t*-test (*n* = 6 mice). **(F)** Representative images of sagittal brain slices from adult mice injected in the lateral ventricles with 3 μl of PHP.eB CaMKII-NLS-GFP-Cre, stained for Hoechst (blue) and anti-GFP (green). Images were acquired at 0.30 mm,−0.90, and−2.18 mm from Bregma (*n* = 3 sagittal brain slices). Scale bar, 200 μm. **(G)** Image representing a sagittal cortical brain slice from an adult mouse injected in the lateral ventricles as described in panel E, stained for Hoechst (blue), GFP (green), and NeuN (red). White squares indicate the brain areas analyzed for the virus spread, i.e., the primary motor cortex (M1), the primary somatosensory cortex (S1), and the hippocampus (HP). **(H)** The virus spread across M1 (*left*), S1 (*middle*), and HP (*right*) is expressed as the ratio of the volume of GFP+ cells over the volume of NeuN+ cells. Points are fit with spline interpolation.

We built rAAV particles to specifically induce REST deletion in cortical and hippocampal glutamatergic neurons. To target the cellular population of interest and to overcome the limited AAV cargo capacity (4.7 kb), we used the CaMKII minimal promoter (370 bp) (Dittgen et al., 2004), which is the minimal sequence of the promoter required to start transcription properly but maintaining cellular specificity (Bedbrook et al., 2018). We cloned the minimal promoter sequence and the Cre/ΔCre sequences fused with a nuclear GFP in the pAAV CAG-NLSx2-GFP plasmid (Figure 1A, *left*). We subsequently verified whether the minimal promoter sequence was sufficient to achieve the expression of the downstream genes with the expected cellular specificity. To do this, we packaged the CaMKII-NLSx2-GFP into AAV1/2 vectors, delivered them through stereotaxic injections in the cerebral cortex of 2-month-old REST floxed mice and, 4 weeks later, performed imaging on brain slices processed for immunohistochemical analysis (Figure 1A, *right*). We analyzed the targeting specificity, calculated as the number of GFP+ cells over the total number of cells, and the penetrance, calculated as the total number of cells positive for cell-specific markers that were also GFP+. Figure 1B shows representative images of brain slices from injected mice, stained with anti-GFP antibodies together with anti-CaMKII antibodies to label excitatory neurons, anti-GAD67 for inhibitory neurons, anti-GFAP for astroglia, and anti-Iba1 for microglia. The CaMKII minimal promoter exhibited a good specificity (mean ± SD: 58.47 ± 3.08%) for CaMKII+ cells and a very low expression in GAD67+ (mean ± SD: 2.63 ± 0.70%), GFAP+ (mean ± SD: 0.46 ± 0.41%), and Iba1+ cells (mean ± SD: 0.90 ± 0.20%) (Figure 1C). On the other hand, the penetrance of the CaMKII minimal promoter showed a good coverage of CaMKII+ cells (mean ± SD: 68.33 ± 4.97%), a low coverage of GAD67+ cells (mean ± SD: 22.80 ± 7.41%), and a minimal penetrance of GFAP+ (mean ± SD: 1.66 ± 1.45%) and Iba1+ cells (mean ± SD: 4.66 ± 0.95%) (Figure 1D).

Once characterized the CaMKII minimal promoter, we packaged our constructs into PHP.eB vectors that provide improved transduction efficiency, higher diffusion throughout the tissue, and the ability to cross biological barriers such as the blood–brain barrier (BBB) (Chan et al., 2017). To optimize AAV delivery to the brain and minimize infection of peripheral tissues, we administered AAVs in the lateral ventricles leveraging on the ability of the PHP.eB capsid to cross the blood–cerebrospinal fluid barrier. We injected 3 μl of virus in each lateral ventricle of 2-month-old REST floxed mice and verified the efficiency of the CaMKII-driven REST inactivation by monitoring the levels of REST mRNA in the hippocampus by RT-qPCR 4 weeks later. Indeed, a significant decrease of REST mRNA was observed in mice injected with Cre with respect to the control mice transduced with ΔCre, consistent with REST deletion in the large subpopulation of excitatory neurons (Figure 1E). We then performed imaging experiments and showed that we were able to reach a good penetrance of the cortex and the hippocampus (Figure 1F). We further characterized the virus spread in the transduced brain areas, namely, motor and somatosensory cortices and dorsal hippocampus (Figure 1G) and found that the percentage of the volume of transduced GFP+ cells over the volume of NeuN+ cells across the three selected areas could be described by a bell-shaped curve. The highest point of the curve, corresponding to the maximum virus transduction in that area, was 31.73% in the primary motor cortex (Figure 1H, *left*), 59.11% in the primary somatosensory cortex (Figure 1H, middle), and 86.59% in the dorsal hippocampus (Figure 1H, *right*). The ventral portion of the brain was not efficiently transduced. No differential effects in astrogliosis or microgliosis were observed after transduction with AAVs encoding either active or inactive Cre (Supplementary Figure 1).

### 3.2 CAMKII-REST-cKO mice do not show major behavioral alterations

**Figure 2:**
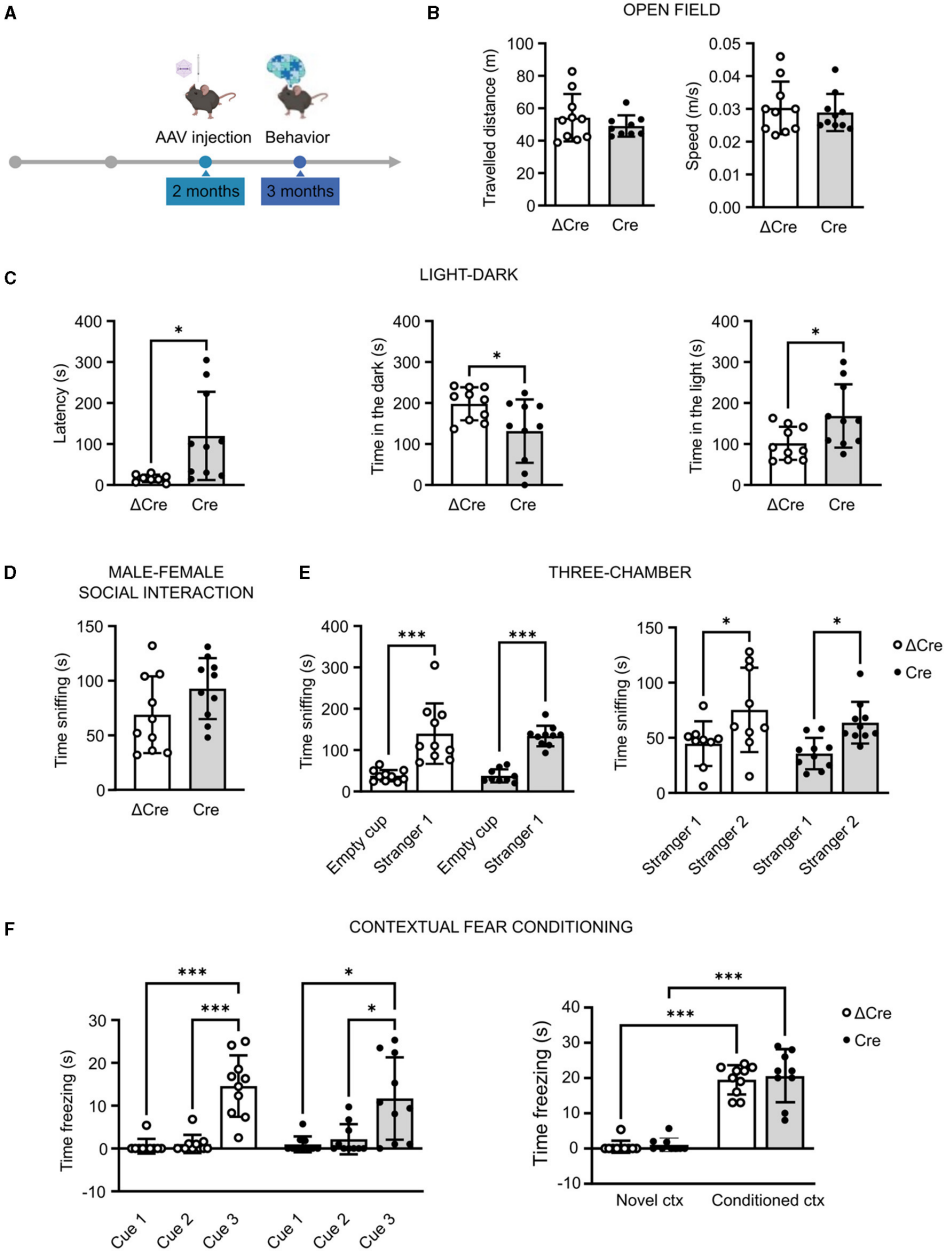
REST knockout in forebrain excitatory neurons does not alter motor, cognitive, and social behavioral performances. **(A)** Timeline of behavioral experiments. **(B)** Open field test: traveled distance (*left*) and mean speed (*right*). **(C)** Light–dark box test: latency to enter the dark compartment (*left*), time in the dark compartment (*middle*), and time in the light compartment (*right*). **(D)** Time sniffing the female subject in the male–female social interaction test. **(E)** Three-chamber test: time sniffing the non-social stimulus “Empty cup” or the cup with the social stimulus “Stranger-1” (*left*) and time sniffing the cup with the familiar social stimulus “Stranger-1” or the novel social stimulus “Stranger-2” (*right*). **(F)** Contextual fear conditioning: freezing time during the conditioning phase (*left*) and freezing time during the contextual conditioning (*right*). All data are expressed as means ± SD (*n* = 10 mice for both CaMKII-NLS-GFP-ΔCre and CaMKII-NLS-GFP-Cre). *p < 0.05, ***p < 0.001; unpaired Student's *t*-test or Mann–Whitney's *U*-test **(B–D)**; two-way ANOVA/Tukey's tests **(E, F)**.

REST is involved in several diseases associated with cognitive dysfunctions. For this reason, we performed a battery of behavioral tests, assessing general motor abilities (open field), anxiety (light–dark test), sociability (male-female social interaction and three-chamber test), and cognitive abilities (contextual fear conditioning), in 3-month-old REST floxed mice previously injected with either PHP.eB CaMKII-NLS-GFP-Cre or with PHP.eB CaMKII-NLS-GFP-ΔCre as a transduction control (Figure 2A). Concerning the locomotor activity, neither the traveled distance (ΔCre, mean ± SD: 54.18 ± 14.63; Cre, mean ± SD: 49.07 ± 6.57) nor the speed (ΔCre, mean ± SD: 0.030 ± 0.008; Cre, mean ± SD: 0.028 ± 0.005) revealed significant differences between ΔCre and Cre mice (Figure 2B).

In the light–dark test, performed to specifically evaluate the anxiety levels, Cre mice appeared less anxious than ΔCre mice. Indeed, Cre mice showed a higher latency to enter the dark compartment (ΔCre, mean ± SD: 16.26 ± 9.02; Cre, mean ± SD: 119.8 ± 107.8; *p* = 0.016), less time spent in the dark compartment (ΔCre, mean ± SD: 198.2 ± 40.29; Cre, mean ± SD: 131.5 ± 77.35; *p* = 0.026), and more time spent in the light compartment (ΔCre, mean ± SD: 101.8 ± 40.29; Cre, mean ± SD: 168.5 ± 77.35; *p* = 0.026) than ΔCre mice (Figure 2C). The social competence did not show statistically significant alterations in the male–female social interaction test (ΔCre, mean ± SD: 68.90 ± 35.14; Cre, mean ± SD: 9‘2.80 ± 27.89) (Figure 2D). In the three-chamber test, both ΔCre and Cre mice showed more interest in exploring the social stimulus with respect to the non-social one (ΔCre, mean ± SD: empty cup 38.00 ± 13.92, stranger-1 139.9 ± 73.03, *p* < 0.0001; Cre, mean ± SD: empty cup 37.67 ± 15.99, stranger-1 134.00 ± 24.95, *p* < 0.0001) and more interest in exploring the novel social stimulus than the familiar one (ΔCre, mean ± SD: stranger-1 44.67 ± 20.28, stranger-2 75.33 ± 38.26, *p* = 0.0224; Cre, mean ± SD: stranger-1 35.70 ± 14.21, stranger-2 63.70 ± 18.87, *p* = 0.0285) (Figure 2E). The cognitive hippocampal functions assessed through the contextual fear conditioning test did not reveal overt deficits. Both experimental groups were successfully conditioned (Figure 2F, *left*) and successfully associated the aversive stimulus with the context (Figure 2F, *right*) but with no significant differences in the time spent freezing in the conditioned context (ΔCre, mean ± SD: novel context 0.54 ± 1.70, conditioned context 19.50 ± 4.14, *p* < 0.0001; Cre, mean ± SD: novel context 1.06 ± 1.93, conditioned context 20.56 ± 7.33, *p* < 0.0001).

### 3.3 CAMKII-REST-cKO mice have unchanged seizure propensity in response to a single convulsive dose of PTZ

**Figure 3:**
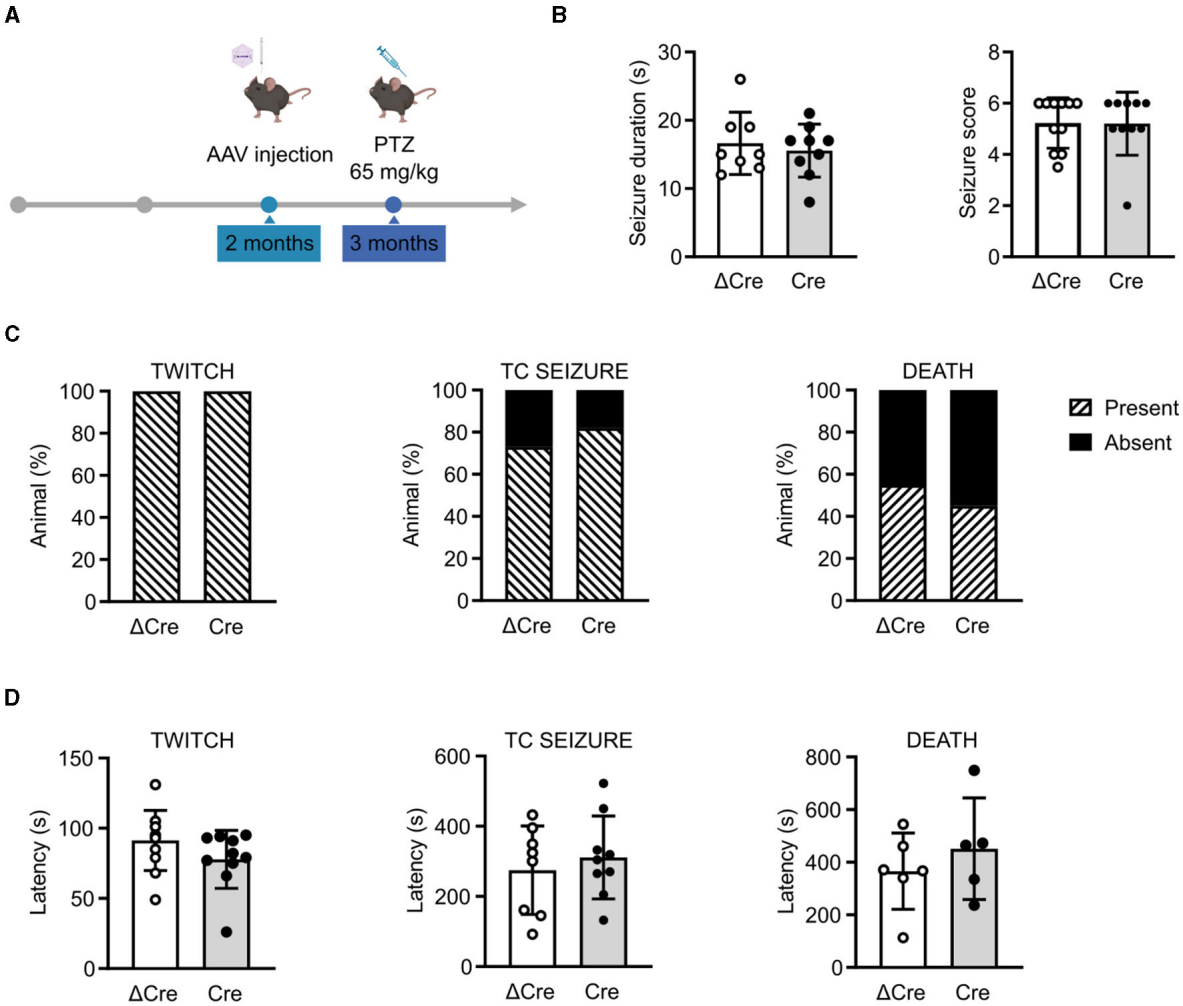
CaMKII-REST-cKO mice have a normal seizure propensity in response to a single convulsive dose of PTZ. **(A)** Timeline of the acute seizure experiment consisting of a single administration of PTZ (65 mg/kg). **(B)** Seizure duration (*left*) and seizure score (*right*) of the two experimental groups during the test. **(C)** Occurrence of myoclonic twitch (*left*), tonic-clonic (TC) seizure (*middle*), and death (*right*). **(D)** Latencies to myoclonic twitch (*left*), TC seizure (*middle*), and death (*right*). Data are expressed as means ± SD (*n* = 11 CaMKII-NLS-GFP-ΔCre mice; *n* = 10 CaMKII-NLS-GFP-Cre mice). *P* > 0.05; unpaired Student's *t*-test or Mann–Whitney's *U*-test **(B, D)**; Fisher's exact test **(C)**.

To investigate the role played by REST in brain hyperexcitability and seizures, we induced acute seizures in 3-month-old REST floxed mice injected with either PHP.eB CaMKII-NLS-GFP-ΔCre or PHP.eB CaMKII-NLS-GFP-Cre, with a single intraperitoneal injection of PTZ (65 mg/kg) (Figure 3A). Animals were videotaped and analyzed for seizure duration and severity score (Figure 3B), occurrence of the first myoclonic twitch, tonic-clonic seizure, and death (Figure 3C), and latency to the first myoclonic twitch, tonic-clonic seizure, and death (Figure 3D). No differences were observed in response to a single convulsive dose in seizure duration (ΔCre, mean ± SD: 16.63 ± 4.56; Cre, mean ± SD: 15.56 ± 3.87), seizure severity score (ΔCre, mean ± SD: 5.22 ± 0.98; Cre, mean ± SD: 5.20 ± 1.22), the occurrence of the first myoclonic twitch (ΔCre, present: 100% absent: 0%; Cre, present: 100% absent: 0%), the occurrence of tonic-clonic seizure (ΔCre, present: 73% absent: 27%; Cre, present: 82% absent: 18%), occurrence of death (ΔCre, present: 55% absent: 45%; Cre, present: 45% absent: 55%), latency to the first myoclonic twitch (ΔCre, mean ± SD: 91.27 ± 21.42; Cre, mean ± SD: 77.80 ± 20.58), latency to the tonic-clonic seizure (ΔCre, mean ± SD: 274.8 ± 125.9; Cre, mean ± SD: 311.0 ± 118.0), and latency to death (ΔCre, mean ± SD: 365.8 ± 145.1; Cre, mean ± SD: 451.2 ± 193.1).

### 3.4 CAMKII-REST-cKO mice show a higher survival rate in the kindling paradigm

**Figure 4:**
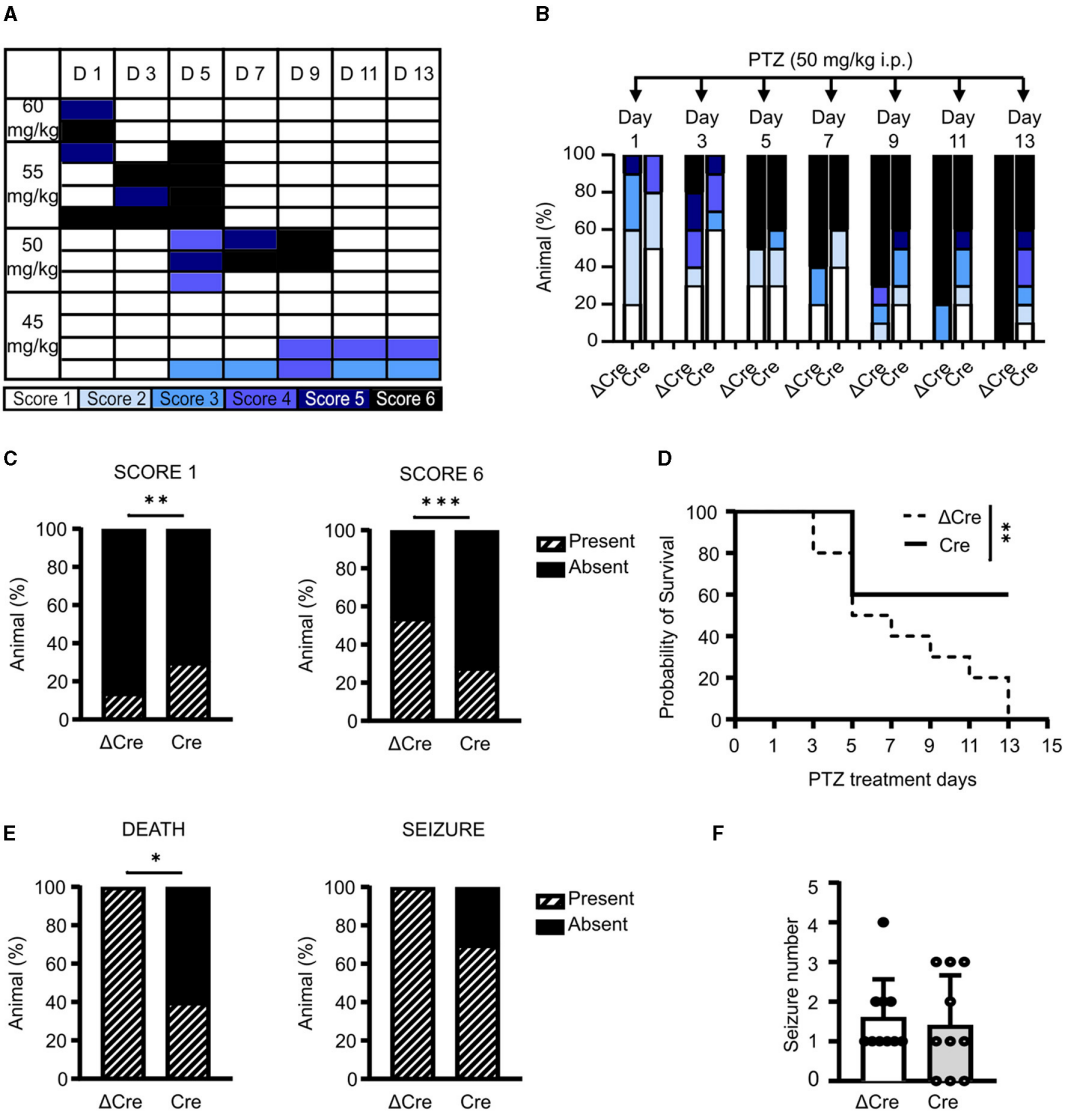
CaMKII-REST-cKO mice show less severe seizures and a higher survival rate in the PTZ kindling paradigm. **(A)** Setting of PTZ dosage for the chemical kindling protocol. The phenotype shown by the tested animals is represented according to the following color code: immobility (white), twitch (light blue), Straub's tail (cyan), mild seizure (blue), severe seizure (dark blue), and death (black). **(B)** Occurrence of the various seizure scores during the defined chemical kindling protocol consisting of PTZ injections (50 mg/kg) on alternate days for 14 days. **(C)** Relative frequency of occurrence of the mildest (Score 1; *left*) and most severe (Score 6; *right*) seizures in the two experimental groups during the chemical kindling protocol. **(D)** Probability of survival of the two experimental groups during the chemical kindling protocol. **(E)** Occurrence of death (*left*) and seizure (*right*) during the chemical kindling protocol. **(F)** Number of seizures experienced by the animals during the chemical kindling protocol. Data are expressed as means ± SD (*n* = 10 mice for both CaMKII-NLS-GFP-ΔCre and CaMKII-NLS-GFP-Cre). *p < 0.05, **p < 0.01, ***p < 0.001; Fisher's exact test **(C, E)**; Mantel–Cox test **(D)**.

Since a single administration of convulsant may not be able to elicit sufficiently fast transcriptional responses to affect the concomitant epileptic behavior, we evaluated the effects of REST knockout in a chronic condition of brain hyperexcitability by performing a pharmacological kindling protocol. As the first step, we defined the optimal PTZ dose to induce a consistent behavioral seizure phenotype. To this aim, we treated various subgroups of 3-month-old REST floxed mice, previously injected with PHP.eB CaMKII-NLS-GFP-ΔCre, with four PTZ doses (60, 55, 50, and 45 mg/kg). Animals were intraperitoneally injected on alternate days for 2 weeks with each dose and scored accordingly to the manifested seizure phenotype: immobility (*white*, Score 1), twitch (*light blue*, Score 2), Straub's tail (*cyan*, Score 3), mild seizure (*blue*, Score 4), severe seizure (*dark blue*, Score 5), and death (*black*, Score 6). As depicted in Figure 4A, 50 mg/kg is the dose that induced the development of a generalized seizure phenotype in a gradual fashion, over more than a week. Once the proper sub-convulsive dose is defined, we performed the same protocol on 3-month-old REST floxed mice injected with either PHP.eB CaMKII-NLS-GFP-ΔCre or PHP.eB CaMKII-NLS-GFP-Cre. Figure 4B displays the occurrence of a given score in each experimental group, and Figure 4C represents the relative frequency distribution of scores, and Cre-transduced mice showed a significantly milder phenotype during the PTZ kindling protocol. Indeed, when we considered the relative frequency of the lowest (Score 1) and of the highest (Score 6) seizure score, Cre mice were interested in a significantly higher occurrence of mild events (30 vs. 14% of ΔCre mice) and a significantly lower occurrence of potentially lethal tonic-clonic seizures (28 vs. 54% of ΔCre mice) (Figure 4C).

Indeed, a larger percentage of Cre-transduced animals presented lower scores with respect to ΔCre-transduced controls (ΔCre, mean ± SD: 4.3 ± 1.9; Cre, mean ± SD: 3.2 ± 2.0, *p* = 0.0016). One of the most evident phenotypes that distinguishes ΔCre- and Cre-transduced mice is survival. As shown in Figure 4D, the probability of survival was significantly different between the two experimental groups (*p* = 0.009), with ΔCre mice showing a probability of survival equal to 0 on the last day of the protocol which was still 60% in Cre mice. In line, death occurrence (Figure 4E, *left*) was significantly lower in Cre mice (ΔCre, present: 10 absent: 0; Cre, present: 4 absent: 6; p: 0.010). To support the hypothesis of a milder phenotype in the absence of REST, we looked at seizure occurrence. Even if all ΔCre mice experienced at least one seizure, while 3 out of 10 Cre mice were unaffected, this parameter was not statistically significant (Figure 4E, *right*). Finally, the last parameter evaluated, i.e., the number of tonic-clonic seizures per mouse during the PTZ injection protocol (Figure 4F) was not different between the two groups (ΔCre, mean ± SD: 1.60 ± 0.96; Cre, mean ± SD: 1.40 ± 1.26). Overall, these data support the hypothesis that the genetic deletion of REST from excitatory neurons ameliorates the seizure phenotype.

## 4 Discussion

The role of REST in the onset and development of epilepsy is still not clearly defined and the available experimental evidence provides contrasting results. Given the broad action of REST as an epigenetic master regulator, its contribution to the pathology is complex and may differ depending on the type and stage of the disease, as well as on the type of neural cell considered (i.e., glial cells or neurons). Although multiple neuronal and glial types participate in the epileptogenic process and in triggering seizures, the final common pathway is increased excitability and discharge of excitatory neurons. For this reason, in this study, we set up an *in vivo* model in which REST is selectively deleted in the excitatory neurons of the adult forebrain using a recently engineered floxed mouse line that allows the complete ablation of full-length REST as well as of all its shorter isoforms (Nechiporuk et al., 2016). Indeed, in previous studies, the persistent transcription of truncated forms of REST after Cre-mediated deletion may have confused the interpretation of results (Liu et al., 2012).

A second point we addressed is the time frame of REST-cKO induction. Constitutive REST deletion would generate animals that lack the protein ubiquitously; however, the lack of protein during the developmental period would cause alterations that may bias the results obtained in the adult animal. Indeed, REST plays a role in shaping and fine-tuning the neuronal network wiring and plasticity during the early post-natal period (Yeo et al., 2009; Rodenas-Ruano et al., 2012). Thus, a time-restricted deletion of REST would allow us to discriminate more precisely the impact of REST deletion in the adult brain and in animals that otherwise had normal development. To achieve such time-restricted REST-cKO, we took advantage of Cre-expressing AAVs with an engineered capsid named PHP.eB that allows increased transgene expression with higher virus spread through the brain tissue and the ability to cross the biological brain barriers (Chan et al., 2017).

Although intravenous AAV delivery would represent the best option for targeting virtually the whole brain, there are several drawbacks associated with this injection route. Indeed, the amount of required viral particles is 1–2 orders of magnitude higher than that required for stereotaxic injections (Chan et al., 2017) and the systemic delivery of viral particles implies transduction of peripheral tissues and higher exposure to immune surveillance (Montgomery et al., 2016). For these reasons, we sought an alternative delivery route to target a broad portion of brain tissue, while using lower amounts of viral particles. Since we used an engineered capsid exhibiting exquisite permeability through the BBB, we thought that these modified vectors could easily cross the blood–cerebrospinal fluid barrier. Indeed, upon injection of 3 μl of PHP.eB in each lateral ventricle of 2-month-old mice, we observed a remarkable spreading of the virus, which efficiently targeted both the cortex and the hippocampus. The transduction of a larger brain area allowing a more widespread cKO than that obtained with intraparenchymal injections is critical when performing behavioral investigations.

By exploiting the above-described experimental procedure, we characterized some behavioral traits associated with epilepsy and cognitive disorders in which REST is involved, such as Rett syndrome (Abuhatzira et al., 2007; Tang et al., 2016), X-linked intellectual disability (Tahiliani et al., 2007), and autism (Katayama et al., 2016). More specifically, we investigated anxiety and motor, social, and cognitive abilities. The absence of motor deficits in cKO was expected since alterations of motor performance following REST deletion are not reported in the literature. Regarding the anxiety phenotype, the light–dark test highlighted a less anxious phenotype in REST cKO mice. However, the altered anxiety profile does not impact cognitive and social performances. In fact, both the contextual fear conditioning test and social behavior were not affected by REST deletion. The brain regions that relate to social and cognition largely belong to the limbic system, which is composed of the amygdala, hippocampus, thalamus, hypothalamus, basal ganglia, and cingulate gyrus. In our experimental model, AAV injection targets mainly the hippocampus, therefore leaving REST expression unaffected in the areas that are most involved in the modulation of these behavioral domains (Ko, 2017).

The main focus of our study was to study the role of REST in assessing seizure susceptibility in response to the administration of the convulsant PTZ that induces generalized seizures. PTZ is a non-competitive antagonist of GABA_A_ receptors even if the precise mechanism of action is still not clear (Hansen et al., 2004). Overall, the use of this drug allowed us to obtain a seizure phenotype that was not directly correlated to a specific brain area. We first performed the acute induction of seizures by a single PTZ administration. In this model, we did not detect any difference between Cre- and ΔCre animals. This is in contrast with the study of Liu et al. (2012), which shows that REST-cKO animals subjected to the acute PTZ seizure model were less vulnerable to seizures. However, one substantial difference between our study and the study by Liu et al. (2012) should be highlighted. In Liu et al.'s (2012) study, REST was knocked out in all neurons by using the panneuronal NSE promoter, while in our study, REST deletion was restricted to forebrain excitatory neurons. Moreover, the NSE promoter has been active since embryonic development, and, as discussed above, this may cause developmental alterations that could influence the results obtained in adult mice. By taking into consideration both our data and the data from Liu et al. (2012), we can conclude that REST expression in excitatory neurons is not impacting the acute seizure response, while we cannot presently exclude a contribution of REST expressed by inhibitory neurons.

We subsequently performed a kindling protocol consisting of repeated PTZ injections over 2 weeks, an approach that allows monitoring of the progressive brain response to a seizure-provoking agent. This is particularly relevant when considering the relatively long time required to modulate the activity of the epigenetic machinery, of which REST is a crucial player. Our data show that REST-cKO mice are less susceptible to developing a severe seizure phenotype that eventually leads to death. Indeed, while the total number of seizures was comparable between the two genotypes, REST-cKO mice showed an overall less severe seizure score, observed at all tested time points, and an improved survival rate. These data indicate that REST expression within excitatory neurons is one important factor contributing to modulate seizure susceptibility, especially the most severe seizure phenotypes (McClelland et al., 2011; Liu et al., 2012; Carminati et al., 2020).

To have a complete picture of the complex role played by REST in epilepsy, other cell-type-specific REST-cKO models in inhibitory neurons and glial cells should be characterized. Indeed, it was recently shown that REST contributes to the homeostatic plasticity of inhibitory synapses (Prestigio et al., 2021), an effect that could be relevant in the context of epilepsy. Moreover, the role played by astrocytes in epilepsy cannot be ignored. Under physiological conditions, astrocytes play several homeostatic roles in brain networks, including K^+^ buffering and glutamate uptake which help controlling neural excitability. Interestingly, dysregulation of Kir4.1 channels was linked to several pathologies associated with hyperexcitability (Tong et al., 2014). We and others have found that REST stimulates the transcription of the GLT1glutamate transporter, which in turn favors the membrane exposure of Kir4.1, boosting glutamate uptake and K^+^ buffering and preserving a physiological level of network excitability (Pajarillo et al., 2021; Centonze et al., 2023). Thus, we believe that a detailed characterization of other cell-type-specific REST-cKO models will give more insights into the role of REST in epileptogenesis and seizure generation. Nevertheless, the results of this study highlight the importance of REST as a key regulator of neuronal excitability, underlying the importance of studying the function of this transcription factor in the different neuronal and glial populations. Overall, this study contributes to the understanding of the complex mechanisms underlying epileptogenesis and adds an additional piece to the puzzle of the role of REST in epileptic susceptibility that identifies REST as one potential target for future therapeutic approaches.

## Data availability statement

The raw data supporting the conclusions of this article will be made available by the authors, without undue reservation.

## Ethics statement

The animal study was approved by OPBA San Martino Hospital - Genova. All the experiments were carried out in accordance with the guidelines established by the European Community Council (Directive 2014/63/EU of 15 May 2014) and were approved by the Italian Ministry of Health (Authorization #558/2016-PR and #427/2021-PR).

## Author contributions

GN: Data curation, Formal analysis, Investigation, Methodology, Writing—original draft. CM: Conceptualization, Data curation, Formal analysis, Investigation, Methodology, Supervision, Writing—original draft. AK-R: Investigation, Methodology, Writing—review & editing. TF: Methodology. FC: Conceptualization, Data curation, Investigation, Methodology, Project administration, Supervision, Writing—original draft, Writing—review & editing. FB: Conceptualization, Data curation, Funding acquisition, Project administration, Resources, Supervision, Writing—review & editing.
