# Supplementary material for: Conditional knockout of REST/NRSF in excitatory neurons reduces seizure susceptibility to chemical kindling
Source: Front Cell Neurosci. 2023 Nov 16;17:1267609. doi: 10.3389/fncel.2023.1267609 (PMC10687554; doi:10.3389/fncel.2023.1267609)
Supplement: Supplementary file 1 [file Data_Sheet_1.docx]

Conditional Knockout of REST/NRSF in Excitatory Neurons Reduces Seizure Susceptibility to Chemical Kindling

Giulia Natali^1,2^, Caterina Michetti^1,2^, Alicja Krawczun-Rygmaczewska^1,4^, Thomas Floss^3^, Fabrizia Cesca^1,4*^ and Fabio Benfenati^1,5*^

^1^Center for Synaptic Neuroscience and Technology, Istituto Italiano di Tecnologia, Genova, Italy

^2^Department of Experimental Medicine, University of Genova, Genova, Italy

^3^Helmholtz Zentrum München, Deutsches Forschungszentrum für Gesundheit und Umwelt, Neuherberg, Germany

^4^Department of Life Sciences, University of Trieste, Trieste, Italy

^5^IRCCS Ospedale Policlinico San Martino; Genova, Italy

***Correspondence:**Fabrizia Cesca
[fcesca@units.it](mailto:fcesca@units.it)

Fabio Benfenati
[fabio.benfenati@iit.it](mailto:fabio.benfenati@iit.it)

**SUPPLEMENTARY FIGURE**

**
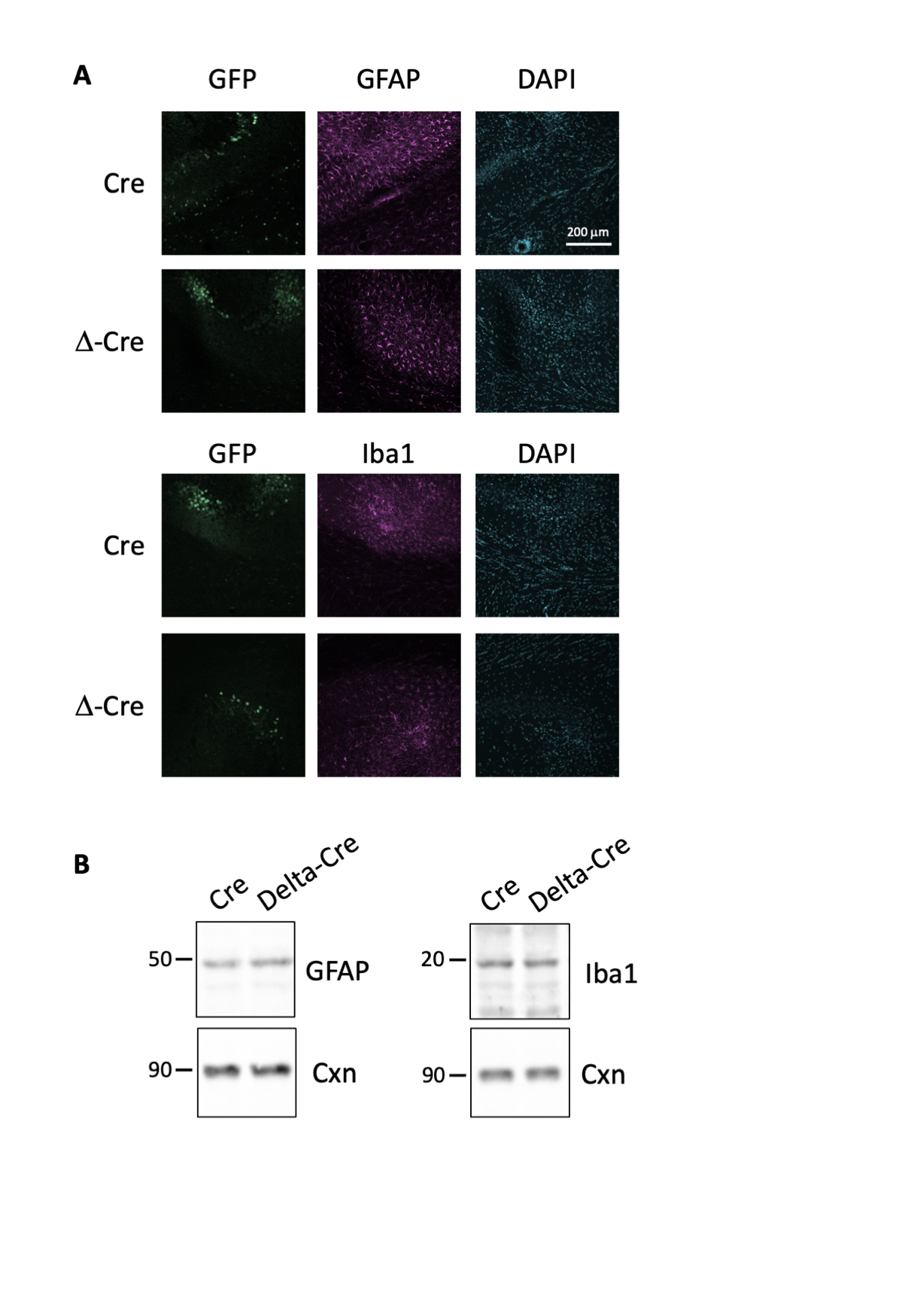
**

**Supplementary Figure 1. Transduction with AAVs encoding either active or inactive Cre does not elicit differential effects in astrogliosis or microgliosis.**

**A.** Cortico-hippocampal slices from mice injected with either Cre (upper rows) or Delta-Cre (lower rows) viruses were stained with anti-GFAP and anti-Iba1 antibodies to monitor for astrogliosis and microglia activation, respectively. Green channel, GFP-viruses; Blue channel, DAPI-stained nuclei; Magenta channel, GFAP or Iba1. Scale bar, 200 µm. **B.** Hippocampal tissues from Cre and Delta-Cre injected mice were lysed and analysed by western blot analysis using anti-GFAP and anti-Iba1 antibodies. Calnexin immunoreactivity was used to control for equal loading.
